# Effect of Extracts of *Terminalia chebula* on Proliferation of Keratinocytes and Fibroblasts Cells: An Alternative Approach for Wound Healing

**DOI:** 10.1155/2014/701656

**Published:** 2014-02-26

**Authors:** Dolly Singh, Deepti Singh, Soon Mo Choi, Sun Mi Zo, Rakesh Mohan Painuli, Sung Won Kwon, Sung Soo Han

**Affiliations:** ^1^Department of Nano, Medical & Polymer Materials, College of Engineering, Yeungnam University, 280 Daehak-Ro, Gyeongsangbuk-do 712749, Republic of Korea; ^2^YU-ECI Medical Research Center, Yeungnam University, Gyeonsanbuk-do 712749, Republic of Korea; ^3^H.N.B. Garhwal University (A Central University), Garhwal, Srinagar, Pauri, Uttarakhand 246001, India; ^4^College of Pharmacy, Seoul National University, Seoul 151-742, Republic of Korea

## Abstract

*Terminalia chebula* is one of the traditional medicines used in the treatment of many diseases. In the present work, different concentrations of various organic and aqueous extracts (solvent-free) of *T. chebula* were tested on fibroblast (L929) and keratinocytes cells to evaluate its biocompatible concentration by using MTT and live-dead viability/cytotoxic assay. These extracts were found to be effective in decreasing the ammonia accumulation in the media, thereby reducing its toxic effect on cells. DPPH assay further confirmed the free-radical scavenging ability of the extracts which increased with the increase in concentration of each extract. Cell proliferation/apoptosis, cytoskeletal structure, and ECM production were further evaluated by live-dead assay and phalloidin/cytokeratin staining, respectively. The cytoskeletal structure and ECM secretion of the cells treated with extracts showed higher cellular activity in comparison to control. In conclusion, we have demonstrated the effect of these extracts of *T. chebula* on both types of skin cells and optimized concentration in which it could be used as a bioactive component for wound healing applications by increasing cell proliferation and decreasing free-radical production without affecting the normal cellular matrix. It can also find applications in other therapeutics applications where ammonia toxicity is a limiting factor.

## 1. Introduction

Chronic wounds, often associated with arterial and venous ulcers and diabetic and pressure sores, is an area of major concern as the direct or indirect costs are substantial and reaching far beyond the costs of hospitalization and physicians. Besides chronic wounds, the degradation of skin extracellular matrix (ECM) involving both dermal and epidermal layers due to aging factors is also a major concern for dermatologists. There are numerous physiological changes that lead to a proteolytic degradation of network of fibers from ECM resulting in scarring of skin tissue especially the dermal region [1]. Generally in humans, scarring of skin and other body parts is due to the excessive proliferation and production of fibroblast cells and its ECM. Hence the fibroblast cells are major targets in therapeutic drug design, where the drug can maintain, control, and balance fibroblast proliferation and apoptosis while maintaining the production and degradation of matrix [2]. Prevalent treatment is using intralesional corticosteroid injections and/or surgery where both patient and clinician do not meet the satisfactory outcome [3, 4]. Lately, scientists and researchers have conducted and formulated new drugs that normalize the morphology of connective tissue during repair by regulating the production of cell proliferation, synthesis, and reduction of extracellular matrix [2].

Few of the natural compounds like phenolic acids and flavonoids that are common in plant families and are found in excessive amounts in vegetables, fruits, cocoa, grains, tea, coffee, beer, and red wine [5, 6] have been known to be bioactive with medicinal properties. The dietary phenolic acids and flavonoids have been investigated by scientists for decades and are generally anti-inflammatory, antioxidant, antidiabetic, and anticarcinogenic in nature [2], hence recommended by many clinicians and dieticians as their dietary intake can improve health and prevent the body from oxidative damage, cancer, and cardiovascular diseases [5, 6]. Connective tissues are protected and prevented from degrading action of elastases by bioflavonoids which bind to elastin, thereby inhibiting the mechanism for enzymatic action on tissues and its components [1]. The antioxidant and free-radical scavenging property of flavonoids can be attributed to the high mobility of the electrons in the benzenoid nucleus and are well known for enhancing the wound healing process [7]. These properties can be easily utilized for skin related ailments as reducing the production of free radicals prevents the damaging of skin cells structure and functions.

Substantial evidence of the use of herbal and medicinal plants in both western and eastern countries dates back to some 60,000 years [8], in which one of the plants recorded was *Terminalia chebula (T. chebula)* Retz, belonging to the family Combretaceae. The plant exhibited various therapeutic uses due to the presence of various phytochemicals in different plant parts [9]. The fruit of this plant is reported to possess the phytoconstituents responsible for antimicrobial [10], antioxidant [11], antiviral [12], anticarcinogenic [13], hypocholesterolemic [14], radio-protective [15], antispasmodic, and antipurgative [16] like gallic acid, ellagic acid, and corilagin [17]. Cytotoxic properties of this plant have also been reported previously by various groups [18, 19]. Leaves and fruits of this medicinal plant have specifically contributed to wound healing management with a decreasing rate of epithelialization and an improved rate of contraction during skin healing [10, 11]. The fruits of *T. chebula* have been reported to have a high content of phenolic compounds and flavonol glycosides and other phytoconstituents responsible for its various therapeutic activities [20].

In this study, we have explored the biocompatibility concentration of various extracts of *T. chebula* on keratinocytes and L929 fibroblasts cell lines. Ammonia and free-radical scavenging abilities of these extracts were also evaluated using ammonia and DPPH assay. Extracts were tested for the presence of bioactive components responsible for all the activity using HPLC and FT-IR spectrophotometric studies.

## 2. Materials and Methods

### 2.1. Materials

*T. chebula* fruits (voucher no. TERCHE 0413 deposited at plant herbarium YNUH, Republic of Korea) were collected from HNB Garhwal University (a central university) campus, Srinagar, Garhwal, India. Organic solvents like ethyl acetate, acetone, and methanol and reagents such as 2,2-Diphenyl-1-(2,4,6-trinitrophenyl) hydrazyl (DPPH), Dulbecco's modified Eagle's medium (DMEM), 3-(4,5-dimethylthiazol-2-yl)-2,5-diphenyltetrazolium bromide (MTT), glutaraldehyde (50%), trypsin-EDTA, fetal bovine serum, penicillin-streptomycin, nystatin, phalloidin and DAPI, sodium nitroprusside, sodium hydroxide, disodium hydrogen orthophosphate, sodium hypochlorite, 1N sulfuric acid, and sodium tungstate were purchased from Sigma Aldrich (Yongin, South Korea). Monoclonal anticytokeratin and TRITC labeled secondary-IgG antibody was obtained from Sigma Aldrich (St. Louis, U.S.). Live/Dead viability/cytotoxicity Kit was purchased from Abcam (Cambridge, U.K.). Keratinocytes cell was obtained from American Type Culture Collection (ATCC) (Manassas, VA, USA) and Fibroblasts cell line (L929) was procured from Korean Cell Bank (Seoul, South Korea).

### 2.2. Methods

#### 2.2.1. Preparation of Extracts

Extracts were prepared using soxhlet extraction method, in which solvents of increasing polarity index (ethyl acetate/acetone/methanol/water) were used. 40 g of dried fruit powder of *T. chebula* was used for the extraction procedure. The solvent-free dry extract (S1: ethyl acetate; S2: acetone; S3: methanol; S4: aqueous) was obtained by evaporating the solvent using rota-evaporator and extracts were lyophilized. Dried extract was kept at 4°C until further use.

#### 2.2.2. Effect of *T. chebula* Extracts on Fibroblasts and Keratinocytes *In Vitro*

Biocompatibility of various organic (S1–S3) and aqueous (S4) extracts was tested on fibroblast and keratinocytes cells. Both cell lines used were previously cultured and maintained in 90% DMEM media substituted with 10% fetal bovine serum and 1% antibiotic for 24 h–48 hrs and maintained throughout the experiments in the same culture conditions. For cytotoxic study, cell monolayer was trypsinised by the usual method to obtain single cells. Briefly, after removing excess of media, the cell layer was gently washed with phosphate buffer saline PBS (0.1 M pH7.0) in order to completely remove the traces of media. Later, to remove adherent cell layer, 500 *μ*L of trypsin-EDTA was added and the culture flask was incubated in a CO_2_ incubator for 5 min. After 5 min, single cells were collected by adding fresh media to the flask. L929 (1 × 10^5^) and keratinocytes (1.2 × 10^5^) were seeded in a 24 well-plate. Cells were incubated with 1 mL DMEM containing various concentrations (10 *μ*g/mL, 50 *μ*g/mL, and 100 *μ*g/mL) of different solvent-free extracts (S1–S4) [21, 22]. Plates were then incubated at 37°C in 5% CO_2_ incubator till the experiment was completed.

#### 2.2.3. Cytoprotective Activity-MTT Assay

MTT assay is a test to check metabolic activity of proliferating cells in *in vitro* conditions. After 24 hrs of incubation with various extracts, media were removed, 500 *μ*L MTT reagent (0.5 mg/mL media) was added in each well and plates were kept for 2-3 hrs in incubator (Day 1). After 3 hrs of incubation, the MTT reagent was removed and 1.5 mL of dimethyl sulfoxide (DMSO) of cell culture grade was added to each well. Intensity of the purple formazon solution was measured at 490 nm in spectrophotometer [23]. Each experiment was performed in triplicates and the same protocol was followed until the completion of the experiment.

#### 2.2.4. Cell Viability Assay

Cell-drug interaction was also evaluated using cell viability assay (live-dead staining kit) over the period of 21 days. From the stock solution, 4 *μ*M of calcein-acetomethoxy (calcein AM-5 *μ*L) and 2 mM of ethidium-bromide (20 *μ*L) were diluted in 10 mL PBS (tissue culture grade). 20–50 *μ*L of working solution was added to each well and plates were incubated for 30–45 min and labeled cells were observed under Nikon fluorescent microscope as per general protocol [23].

#### 2.2.5. Effect of Extract on Cytoskeleton and Marker for Keratinocyte Proliferation

Media were discarded from test wells and washed using phosphate buffer saline containing 1% (w/v) bovine serum albumin (BSA) and 0.1% (v/v) Triton X-100 (surfactant) for 30 min at room temperature. Primary antibody, phalloidin biotin for intractyoplasmic staining used for staining cytoskeleton of L929 was prepared in 1 : 100 dilutions in minimal PBS containing 0.1% BSA. Stain was added, just enough to cover the test wells and kept for overnight incubation at 4°C. Following the incubation, test wells were washed with PBS again containing 0.1% BSA thereafter; FITC labeled secondary antibody was added in 1 : 200 (working solution) dilutions. Plates were wrapped in aluminum foil for 45 min and kept at room temperature. After incubation, test wells were extensively rinsed with PBS containing 0.1% BSA to wash away excess of secondary antibody, repeated 5 times, as per manufacturer's protocol.

For keratin production (marker, ECM of keratinocytes) in *in vitro* conditions (for treated and untreated cells), cells were washed with 1 mL of PBS containing 2% Tween 20 (solution 1). Plates were slowly shaken for 5 min. This solution was removed and the blocking solution (mixture of solution 1 and FBS) was added to each test and control wells. Plates were incubated for 30 min at room temperature. After 30 min, the blocking solution was discarded and working solution of 1° stain (monoclonal anticytokeratin) in PBS (1 : 200) was added. Plates were kept at room temperature for 1-2 hrs, after which it was transferred to 4°C for overnight incubation. TRITC-IgG secondary antibody in PBS (1 : 200) was prepared and added to cells stained with 1° antibody (removed after overnight incubation) and incubated for 30–45 min.

After completion of the staining procedure, all stained cells (phalloidin, cytokeratin) were viewed under fluorescent microscope (Nikon Ti-U) with Green and UV filter and images were recorded.

#### 2.2.6. Estimation of Ammonia

This test was performed to check the accumulation of ammonia over a longer *in vitro* culture period. To test for effect of extract on ammonia production, ammonia free water was used for preparing all required reagents and washing of glass-wares. 1 mg of phenol was dissolved along with 5 mg of sodium nitroprusside in 100 mL of degassed ammonia free water and labeled as solution I. Solution II was prepared by mixing sodium hydroxide (250 mg), 2.16 g of disodium hydrogen-orthophosphate in 10 mL of sodium hypochlorite, and total volume was made to 50 mL. Both solutions were mixed well and tightly capped until further use. The test samples (250 *μ*L) were taken and mixed with 1 N sulfuric acid and 10% sodium tungstate which instantly denatures proteins and sediment by centrifugation at 1700 g for 20 min. The supernatant was immediately collected and mixed with 2.5 mL of both solutions I and II [24, 25]. After complete mixing, tubes were incubated at 37°C for 40 min and the absorbance was recorded at 625 nm. For ammonia a standard same protocol was followed with BSA (as a standard) to obtain a standard graph.

#### 2.2.7. Antioxidant Analysis of Extract

Antioxidant activity was evaluated using DPPH assay [26]. Briefly, 300 *μ*L of extracts (10 *μ*g/mL, 30 *μ*g/mL, 50 *μ*g/mL, 70 *μ*g/mL, and 100 *μ*g/mL) were mixed with 2.7 mL of DPPH (final concentration of DPPH was 2.0 × 10^−4^ M) and mixture was vigorously shaken to ensure proper mixing of the extracts and DPPH reagent. Mixture was then allowed to incubate at room temperature for 30 min and absorbance was observed at *A*_517_ [27]. Mean average value was noted as the experiment was performed in triplicates. Absorbance of DPPH was noted as *A*_*o*_ and IC_50_ was calculated using formula

(1) $$\begin{array}{c} {\text{IC}}_{50}=\left(\frac{A_{o}-A_{e}}{A_{o}}\right)\times 100, \end{array}$$

where *A*_*e*_ is the absorbance of the extracts. Antioxidant potential of all the extracts at each concentration was calculated and is represented as a graph drawn between percentage inhibition versus concentrations.

#### 2.2.8. FT-IR, HPLC, and LC-MS Analysis of Extracts

FT-IR characterization (PerkinElmer Spectrum 100-USA) was done with lyophilized extracts powder (S1–S4). Extract powder was placed on KBr pellet and IR peaks were recorded.

High Performance Liquid Chromatography (HPLC) study was performed using C18 PCX 500 Dionex analytical column (WATER 2414 refractive index detector and 515 HPLC pump) with detection of phytocompounds at 260 nm using UV detector. Working test samples were prepared by dissolving the solvent-free crude extracts in DMSO (1 mg/mL) and 20 *μ*L of these samples was injected into the column keeping total sample run time to 15 min. 0.1 M KCL, 0.05 M HCL, and 10% acetonitrile were passed through the column as mobile phase [28]. Standard reference compound used was gallic acid as its one of the most important major compounds of *T. chebula*.

LC-MS analysis was performed using inlet method (Perkin Elmer Flexar LC); the column used was ACQUITY BEH C18 (2.1 × 100 mm, 1.7 *μ*m) and conditions were gradient (A: water, 0.1% formic acid, B: acetonitrile, 0.1% formic acid): 5% B (3 min), 5%–>35% B (20 min), 35%–>100% B (2 min), 100% B (7 min) Detector PDA: 254 nm MS: 100–1500 m/z, negative mode.

## 3. Results and Discussion

### 3.1. Cytotoxic Study of Extracts on Fibroblasts and Keratinocytes

**Figure 1:**
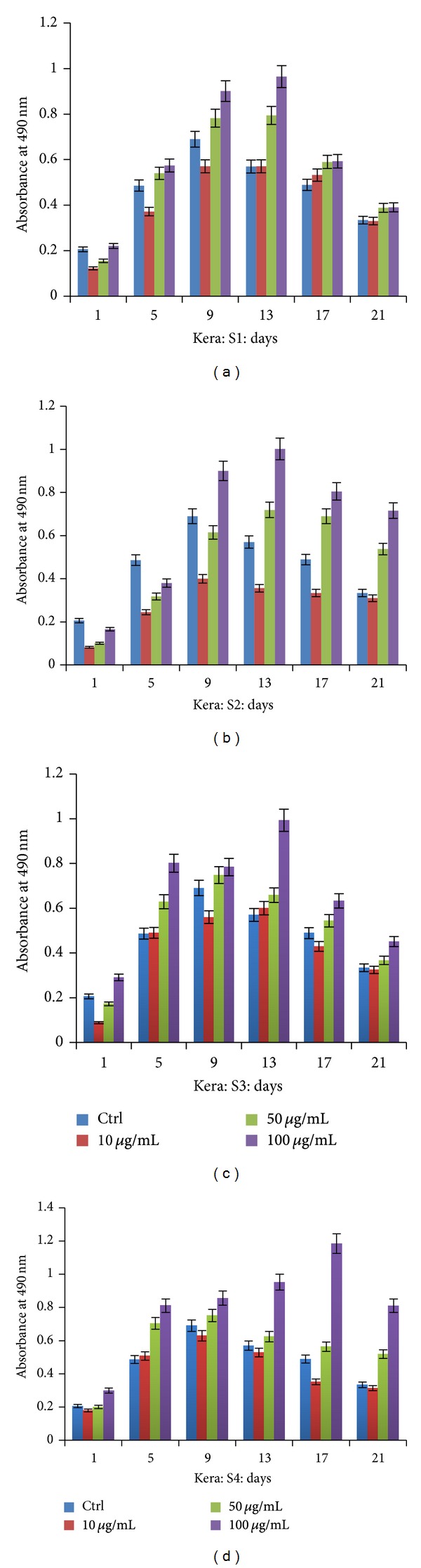
MTT assay performed using different extracts (ethyl acetate S1 (a), acetone S2 (b), methanol S3 (c), and aqueous S4 (d)) on keratinocytes over the period of 21 days.

In this study, we have explored the effect of various extracts of fruits of *T. chebula *on human fibroblasts (L929) and keratinocytes cell lines. Effect of various concentrations of S1–S4 extracts on keratinocytes (Figure 1) was checked using MTT assay which showed that the cells were metabolically more active in the presence of all the extracts in comparison to control (without extracts). Keratinocytes were actively proliferating in the presence of S1 extract (Figure 1(a)) even at a higher concentration in comparison to control; however, there was a gradual decrease in the activity after 2 weeks both in treated as well as untreated cells. Increased concentration of various extracts did not affect the metabolic process of cells, indicating biocompatibility of phytoconstituents present in these extracts. Among all four extracts, S2 (Figure 1(b)) and S4 (Figure 1(d)) showed better results than S1 and S3 (Figure 1(c)) as with increase in concentration of S2 and S4 extracts; a high percentage of proliferating and metabolically active cells were observed even after 21 days of culture, whereas S1 and S3 extracts although did not show cytotoxicity, there was no significant difference between control and treated cells.

**Figure 2:**
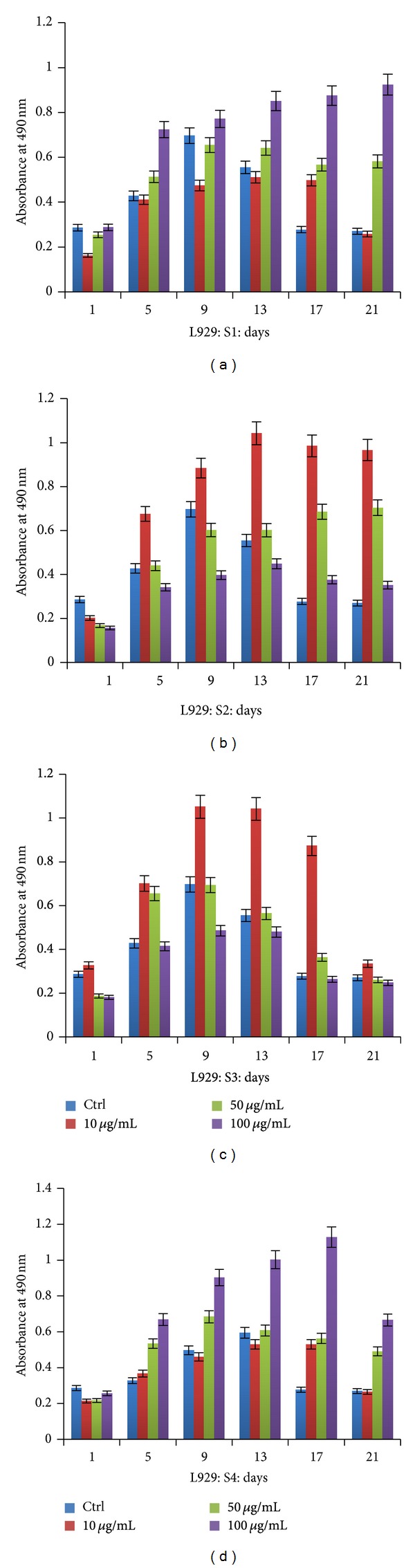
MTT assay performed using different extracts (ethyl acetate S1, acetone S2, methanol S3 and aqueous S4) on L929 fibroblasts over the period of 21 days showing a varying percentage of metabolic activity.

MTT assay performed on L929 fibroblasts showed that cells were metabolically more active at lower concentrations of S2 and S3 but not in higher concentration (Figures 2(b) and 2(c)), whereas S1 and S4 extracts (Figure 2(d)) were found to be effective even at a higher concentration of 100 *μ*g/mL (Figures 2(a) and 2(d)). Even after 21 days of experiment, cells survived and proliferated in treated wells, whereas cells were found to be less metabolically active in control. The biocompatibility study of *T. chebula* showed an increased proliferation of keratinocytes and controlled fibroblasts in the presence of various extracts than those with untreated cells over the period of 21 days. The principle compound known in *T. chebula* extracts is gallic acid which is an active blocker of T-lymphocyte mediated cytotoxicity which in turn blocks the major immunocascade resulting in enhanced cellular functionality. Besides this cytoprotective effect of *T. chebula* extract on inhibitory effect and oxidative stress on cellular aging is well documented [13, 29–31]. However, this is first time different extracts are tested in the *in vitro* system together to identify the optimal concentration that can influence metabolic activity of cells. This difference in rate of proliferation could be exploited in regeneration of tissue in burn cases or other injuries in which controlled proliferation of fibroblasts and more significant proliferation of keratinocytes are required [32].

**Figure 3:**
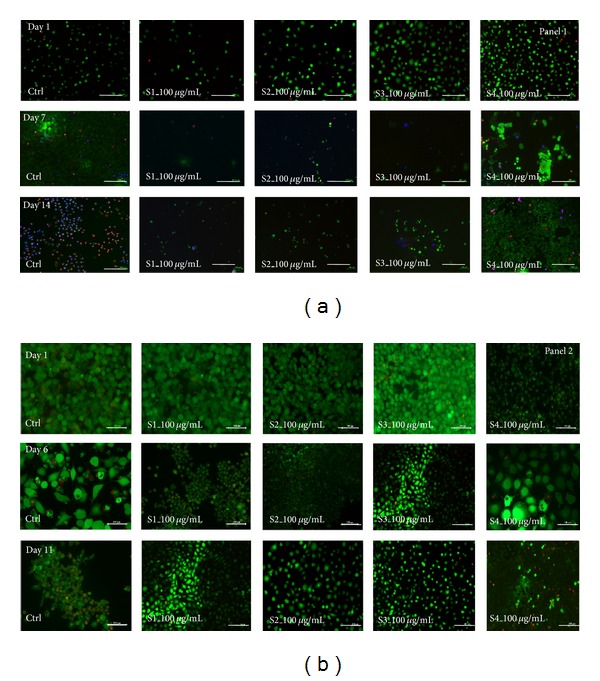
Live/dead cell viability assay of control and treated (*T. chebula* extracts: S1–S4 at a concentration of 100 *μ*g/mL) fibroblasts and keratinocytes cells, respectively (*scale bar represents 100 *μ*m).

For keratinocyte to proliferate it needs a feeder layer and in presence of these extracts fibroblast proliferation was found to be controlled and they could act as substrate for kerationcyte proliferation during initial wound healing process and can later facilitate complete dermal repair in presence of bioactive herbal component [33]; however, fibroblasts have a higher mitotic index than keratinocytes, hence there are a number of techniques applied by the researchers to check overgrowth of fibroblast cells. These techniques include use of mitomycin C treatment or gamma radiation [32]. In this study we have chosen a natural herbal plant and as seen during the live and dead staining (Figure 3) the rate of fibroblast proliferation (Figure 3 panel 1) was significantly slower than keratinocytes (Figure 3 panel 2) which were found to be more active in the presence of S2 and S4 extracts even at a higher concentration, whereas fibroblasts were seen to proliferate slowly in the presence of S2 extract. The mitotically inhibited fibroblast can act as a feeder layer if these cells are cocultured in the presence of this extract, which can be used as the bioactive component that can inhibit/slow down the metabolic rate of fibroblast yet keep cells viable [2] that can aid in two ways: first fibroblast can act as a substrate for keratinocytes in the initial phase of wound healing and later facilitate complete dermal repair [2].

**Figure 4:**
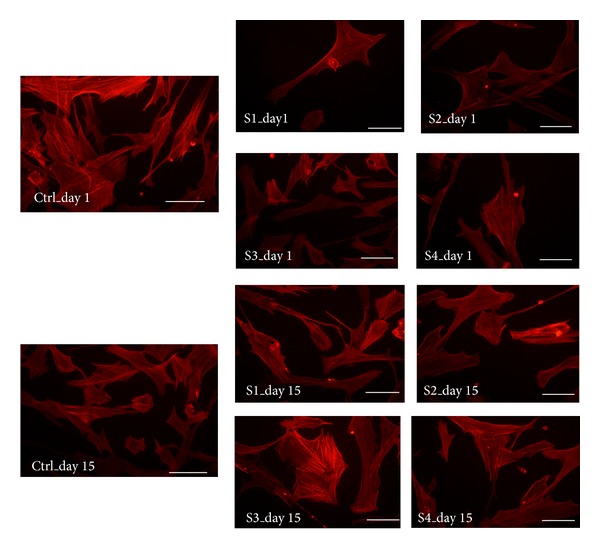
Cytoskeletal staining (phalloidin) images of fibroblast (control and treated with various extracts at different concentrations of *T. chebula* extracts) at different time intervals (scale bar indicated 100 *μ*m).

**Figure 5:**
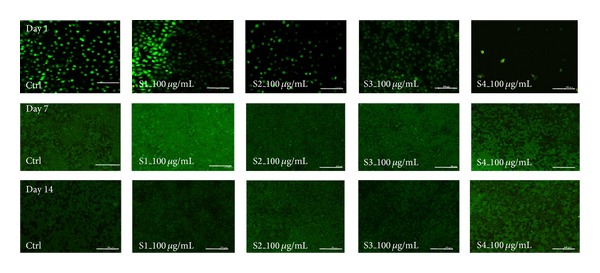
Cytokeratin staining images of keratinocytes (control and treated with various extracts of *T. chebula* extracts) at different time intervals.

Response of both cell lines to various extracts and their percentage change in metabolic activity was different depending on the extract type which can be due to the selective cellular response and affinity to compounds in these bioactive extracts [34]. Treatment of cells with the extracts of *T. chebula* showed a prominent effect on the morphology and growth pattern of both types of cells. Cell proliferation can be controlled in a 2D environment by decreasing cell-cell contact. The cytoskeleton architecture defines the cellular movement and proliferation and checks the effect of extracts on cells; phalloidin staining for fibroblast and cytokeratin staining for keratinocytes were performed, respectively. Fibroblast staining shows highly organized cytoskeletal protein actin (Figure 4) with contractile phenotypes especially in the presence of S1–S4 extracts in comparison to control. Fibroblast has capacity to alter its phenotype depending on the surrounding environment, and especially if found in site of wound, these cells change into collagen producing phenotype which is more contractile than normal cells [35]. Keratinocytes are known to maintain tissue homeostasis and aid in epidermis regeneration following injury. The cytokeratin staining of the keratinocytes shows that keratin expression is maintained by cells cultured in presence of *T. chebula* extracts especially in case of S2–S4 (Figure 5) even till 2 weeks time. These cells were seen to form their own three dimensional ECM with cell-cell communication which is needed in case of skin tissue engineering and skin regeneration. These 3D ECM aids in healing of wounds and remodeling of the injured skin [2]. It is a well-known fact that skin is a dynamic tissue which is made up of different types of cells and fibroblast helps in maintaining the daily wear-tear of the skin; hence it plays an important role during wound healing, especially in burn cases in which even if the skin is remodeled it leaves a scar; hence a bioactive component is needed to ensure the skin remodeling occurs at a faster rate and results in scar free tissue and this could be achieved if the cells are encouraged to secrete their own ECM component with a high proliferation rate [36]. As observed in this experiment the keratinocytes and fibroblast cells both showed higher metabolic activity and successfully formed 3D structure which could show the potentiality of these extracts as bioactive moiety for skin tissue engineering and regenerative medicine.

### 3.2. Determination of Ammonia Reduction

**Figure 6:**
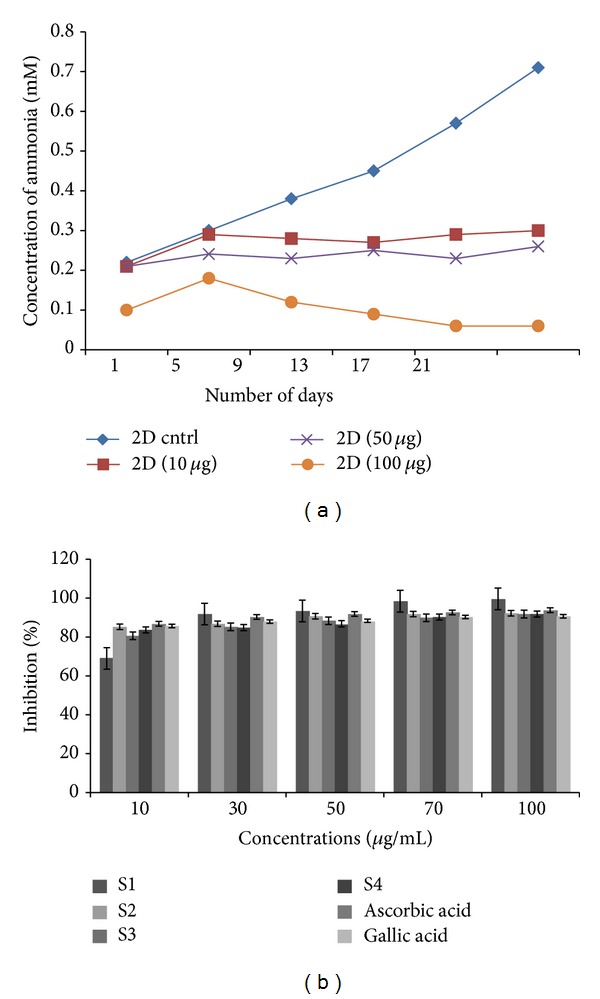
(a) Effect of *T. chebula* extracts on concentrations of ammonia for keratinocytes and (b) DPPH radical scavenging property of S1–S4 extracts, L-ascorbic acid, and gallic acid.

Extended hyperammonaemia condition could result in coma and convulsions according to various research reports [37]. In case of long term cell culture the highly metabolically active cells breakdown the nutrient from the medium for its energy requirement this in turn results in the steady build up of metabolic bi-waste such as ammonia. Ammonia toxicity hinders cellular proliferation as it can alter the metabolic cycle and during antibody production the ammonia build up results in decrease in production of therapeutical agents [38]. Cells incubated at 37°C in the presence of medium containing amino acid specially glutamine and also amino acid metabolism in cells are the main reasons for ammonia accumulation in long-term cell cultures [38]. Amino acid and nitrogen metabolism both in body and in the *in vitro* conditions lead to ammonia build up which is also enabled by the micro-flora present in body. Breakdown of urea by the enzyme urease is one of the important sources of this ammonia but it increases the alkalinity of the surrounding tissue and culture conditions [38, 39], and it is toxic to tissue that is in the process of healing, as in some cases of chronic wound as many as four different types of bacterial growth can be observed at any given time point of healing process [40]. For various mammalian cells elevated ammonia could lead to decreased viability, for example, 3T3 cells inhibition of cell growth was observed when ammonia concentration increased more than 1 mM [25]. Reduction in bacterial toxicity caused by its metabolic end product (ammonia), neoangiogenesis, increased macrophage activity, sufficient fibroblast activity and controlled enzymatic mechanism help in reducing the pH at the site of wound, thereby enhancing the healing process [41–45]. The medium was collected and analyzed for ammonia using standard Indo-phenol reaction. The absorbance of the test product was read at 625 nm and ammonia standard obtained using different concentrations (0, 0.5, 1.0, 1.5, and 2.0 mM) and resultant concentration versus absorbance was plotted to derive the *R* ^2^ value and used further in plotting the ammonia concentration in test sample. In the presence of the extract of *Terminalia chebula* the ammonia concentration was seen to decrease; however in 100 *μ*g dosage the ammonia showed a significant decrease and this value further validates MTT results as the cells were observed to show higher metabolic activity in comparison to untreated and cells cultured in a lower dosage of extracts (Figure 6(a)).

### 3.3. Phytochemical Analysis and DPPH Assay for Free Radical Neutralization

A review among ethanopharmacology has revealed the use of traditional medicine, indigenous plants, as a basis for their herbal therapies [26]. Fruits, cereals, nuts, many spices, and culinary herbs used as daily ingredients in our diet are common major sources of phenolic compounds [2]. Plant's antioxidant or free-radical scavenging ability is related to their phenolic contents, showing the significant presence of these phenolic compounds in various concentrations distributed or localized in plants or their specific parts [6]. Oxidation of metal compounds consumed in trace amount in our diets which initiates the production of free-radicals is scavenged by these phenolic groups which act as metal chelators [26]. DPPH is the assay to check the potential of extracts or proteins as potent antioxidants. DPPH, extracts, L-ascorbic acid, and gallic acid dissolved in 90% methanol were used for the test. L-ascorbic acid and gallic acids were used as positive controls. Phytochemicals extracted in various solvents act as neutralizers or scavengers of free-radicals. With increasing concentration of the extract, discoloration of DPPH radical was observed. Dose-dependent increase in the antioxidant activity was observed in all 4 extracts as well as in L-ascorbic acid and gallic acid. S1–S4 extracts showed better potential to inhibit DPPH radicals than the control (L-ascorbic acid and gallic acid). With the increase in concentration of S1 extract percentage inhibition was found to increase from 69% to 99.61%, inhibiting almost all DPPH radicals present in test solution in comparison to the potent antioxidants like gallic acid and L-ascorbic acid, which ranged from 85.66–90.7% and 86.82–93.8%. S2 (85.27–92.25%), S3 (80.62–91.86%), and S4 (83.72–91.86%) were almost in par with ascorbic acid and gallic acid in reducing the DPPH radicals. The percentage inhibition calculated was recorded and graphs were plotted against concentration versus percentage inhibition (Figure 6(b)). As per the previous study by Blois [26] and Argen et al. [34], IC_50_ value of tests was 100 *μ*g/mL and more whereas in our study even the lowest inhibitory concentration of observed to be 30 *μ*g/mL recording 85% and increased with increase in concentration. Even in comparison to the reference compound (controls) the scavenging property was better than synthetic commercial and earlier reported antioxidants like l-ascorbic acid. This can be attributed to the combinations of phytoconstituents extracted in different solvents of increasing polarity from fruits of the plant rather than leaves or any other parts.

### 3.4. FT-IR, HPLC, and LC-MS Analysis of Extracts

**Figure 7:**
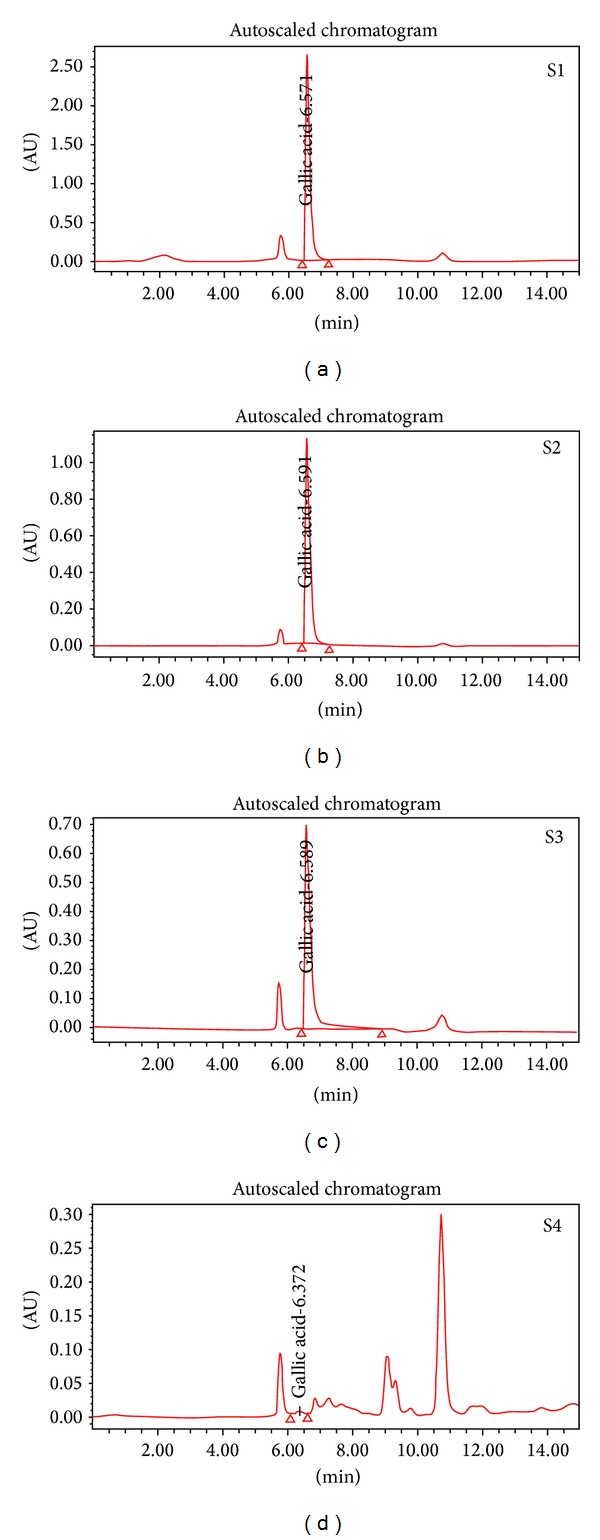
HPLC analysis of *T. chebula* extracts S1 (ethyl acetate), S2 (acetone), S3 (methanol), and S4 (aqueous) detecting gallic acid as one of the major components.

The phytochemicals or bioactive substances' most prominent being alkaloids, flavonoids, tannins, and phenolic compounds initiating positive physiological activity in the human body are attributed to the medicinal value of plants [46]. All four extracts of *T. chebula* had various bioactive components possessing numerous activities. Gallic acid is one of the important compounds of *T. chebula* other than tannic acid which has previously been identified by other researchers [47–50]. Our results were similar to these researchers where gallic acid was identified as one of the compounds in all the extracts. Ethyl acetate and acetone extract of the fruit had gallic acid as a prominent compound where as it was found as in small traces in methanolic and aqueous extracts (Figure 7). Gallic acid used as a reference compound for HPLC analyses had a retention time (R.T) at 6.573 min and similar R.T. was observed in S1 (6.571), S2 (6.591), S3 (6.589), and S4 (6.372) showing the presence of gallic acid (Figure 7). The presence of the higher concentration of other phytosignatures other than gallic acid in S3 and S4 extracts and small quantities in S1 and S2 extracts but could not be identified in the absence of standard compounds.

**Figure 8:**
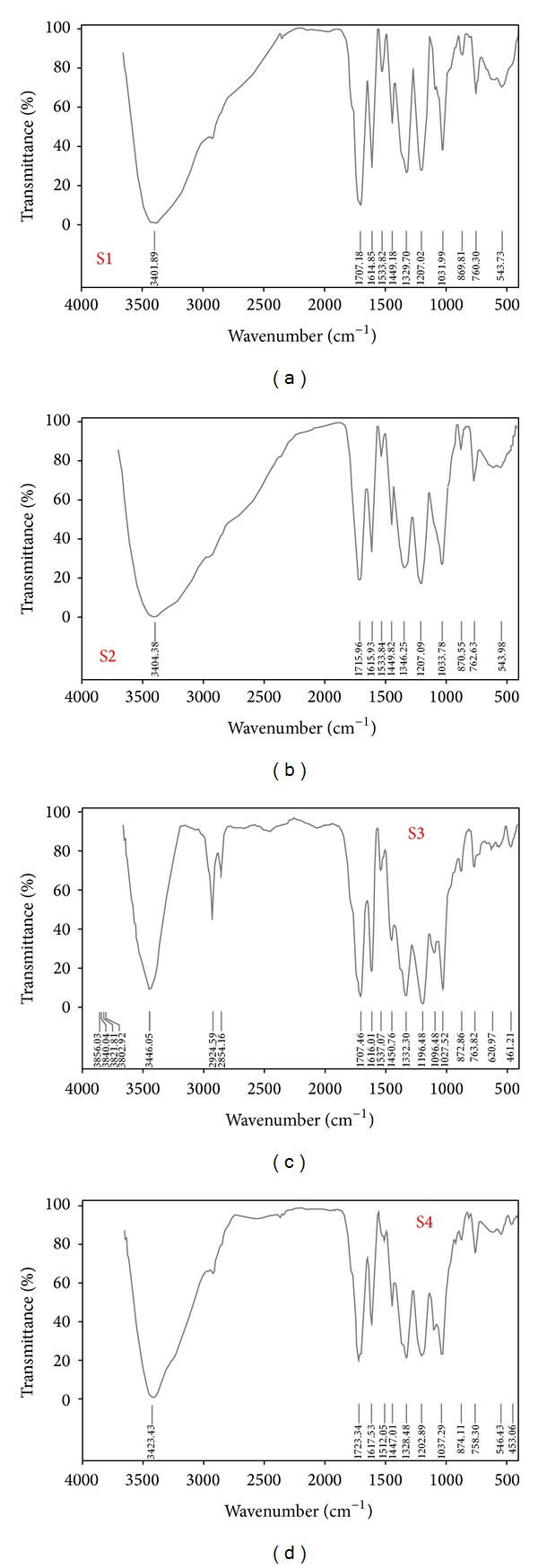
FT-IR graph of *T. chebula* extracts (S1–S4) showing phenols, primary amines, alkyl-methylene, saturated carboxylic acids, and many other peaks revealing the presence of different phytosignatures extracted in organic and aqueous solvents.

From the results obtained by FT-IR study and analysis of the peaks (Figure 8) reveal the presence of high concentrations of phenols, primary amines, alkyl-methylene medium to strong bonded groups, saturated carboxylic acids, multiple broad peaks of ammonium ions, amino acids (zwitterions), N–O nitro-compounds, aromatic meta- and monodisubstituted-benzene, conjugated aromatic groups, bromoalkanes, aliphatic, and aromatic amines (saturated or unsaturated). Apart from the main constituent identified (gallic acid) there are numerous compounds present in the fruit extracts as revealed by FT-IR analysis that can contribute to various activities of the study.

**Figure 9:**
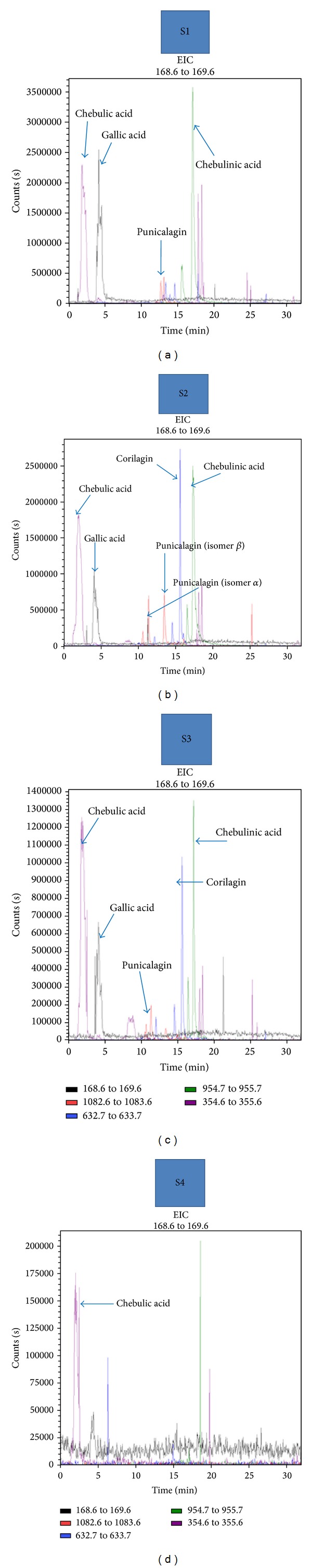
LC-MS spectrum of *T. chebula* showing presence of various phytochemicals.

LC-MS profile of these extracts (Figure 9) shows the presence of a range of phytochemicals like chebulic acid, gallic acid, chebulinic acid, and punicalagin (S1 and S3), and similarly along with these, S2 extract also shows corilagin and punicalagin (isomer *α* and *β*); however these phytochemicals were absent in S4 which could be a reason for the different behaviors of each extract on cells. Chebulic acid is known to inhibit intracellular ROS scavenging especially endothelial cells, similarly punicalagin is also reported to be a potent antioxidant [51]. We believe more than single compounds it is the cocktail of phytochemicals that have synergetic effects on cells which enhance the proliferation rate of cells along with acting as scavengers of free radical and stop ammonia build up in culture conditions.

## 4. Conclusion

Researchers have constantly been focusing on exploring molecules that show a positive effect on overall cellular growth and metabolism and can also help in removing the toxic catabolites. *T. chebula* is a potent antioxidant and is reported to help in improving immunity and control and normalizes digestion, thereby maintaining the sugar level reducing cholesterol as well as being antimicrobial. The cytotoxic study revealed the ability of the extracts to be potent compounds for cellular activity which can be modified and controlled by varying the concentration and can be attributed to the presence of various phytochemicals extracted in different solvents based on their polarity that can be modified according to the nature of injury or wound. The various organic and aqueous extract could be used as a bioactive component for enhancing the rate of wound healing by increasing cell proliferation and increasing free-radical scavenging ability and also in the therapeutics industries in which ammonia accumulation causes a decreased production of the antibodies.
